# Digital epidemiology: what is it, and where is it going?

**DOI:** 10.1186/s40504-017-0065-7

**Published:** 2018-01-04

**Authors:** Marcel Salathé

**Affiliations:** Digital Epidemiology Lab, School of Life Sciences and School of Computer and Communication Sciences, EPFL, Chemin des Mines 9, 1202 Geneva, Switzerland

## Abstract

Digital Epidemiology is a new field that has been growing rapidly in the past few years, fueled by the increasing availability of data and computing power, as well as by breakthroughs in data analytics methods. In this short piece, I provide an outlook of where I see the field heading, and offer a broad and a narrow definition of the term.

Digital Epidemiology has come a long way in the past 10 years. What started as a small and diverse group of researchers from various fields mining the increasing amount of internet data for epidemiological purposes has now turned into a nascent field of its own. The number of scholarly publications, scientific events, and academic groups dedicated to digital epidemiology has grown steadily over the past few years. In these early years of digital epidemiology, its growth has been fueled by the rapid growth of digital data, the widespread penetration of mobile phones and internet usage, and the increasing power of machine learning that is necessary to make sense of the available data. As these underlying developments are continuing, the growth of the field of digital epidemiology will continue alongside with it.

In this short article, my goal is neither to reflect on these past developments, nor to review its growing literature. Rather, it is to do two things: First, to offer something akin to a definition of what digital epidemiology is, and second, to offer an outlook where I see the field heading. Both tasks sound straightforward, but are equally daunting. Defining a field captured by two words is almost guaranteed to cause controversy, as anyone who has tried to define even the simplest daily objects can easily confirm. Nevertheless, I am going to attempt it, in the hope that it can help others identify so far unexplored avenues of digital epidemiology. I’d like to state here clearly and unequivocally that I am neither claiming that this is the only definition one could entertain, nor that I would think that anything outside of that rather strict definition isn’t digital epidemiology. The second challenge - to offer an outlook - is equally daunting, as predictions, when looked at from the future, will have either been wrong or obvious. They should therefore be offered with care.

## A definition of digital epidemiology

The goal of epidemiology, very broadly speaking, is to understand the patterns of disease and health dynamics in populations as well as the causes of these patterns, and to use this understanding to mitigate and prevent disease, and to promote health. The goal of digital epidemiology is exactly the same. So what differentiates (non-digital) epidemiology from digital epidemiology? The broadest definition one can give for digital epidemiology is the following: Digital epidemiology is epidemiology that uses digital data. I expect that this broad and straightforward definition will appeal to many, as it includes any modern approach to epidemiology based on digital sources. I would, however, like to offer an additional and much more narrow definition for digital epidemiology that I personally find more appealing and more thought-provoking, namely the following: Digital epidemiology is epidemiology that uses data that was generated outside the public health system, i.e. *with data that was not generated with the primary purpose of doing epidemiology*.

Here, I need to pause to inject two observations. The first is to state, again, that linguistic definitions are a losing battle - using words to define something will require other words that themselves need definition (e.g. how does one define *“*public health system”, or worse, how does one define “goal”). The second is to underline that I am putting the emphasis on the *purpose* of the data generation, rather than on the *format* of data. I find this definition much more thought-provoking, as it asks us to think creatively about new ways to solve an existing problem. In my experience, the question "what novel data streams generated outside of public health could be leveraged for epidemiological purposes?" forces us to think broadly about the use of new data sources that the digital revolution has brought about. The quality of epidemiology should of course not ultimately depend on whether it uses data that was generated for epidemiological purposes - if anything, it should be much more straightforward to do epidemiology with data created specifically for the task at hand, i.e. to solve an epidemiological problem.

The issue with this definition becomes most visible when we talk about electronic medical records data, collected through regional health information organizations, all payer in-patient billing claims data, and prescription drug databases. Such digital data sources are typically generated as part of the normal digital transformation of business processes, but also to make the data more easily available to epidemiological studies. Whether work on such data sources falls into the narrow definition of digital epidemiology is hard to say. But my main goal for offering a stricter definition here, as stated above, is simply to provoke the identification of so far unexplored avenues and data sources that can potentially enrich epidemiology. Examples from the past for such data sources include search engines, social media services, mobile phones, website access logs - all sources that were generating data without the purpose of doing epidemiology.

## Digital epidemiology - an outlook

The original growth of digital epidemiology was largely fueled by the rapidly increasing amounts of data generated on the internet, particularly also on social media. Google Flu Trends (GFT) was among the earliest well known examples of digital epidemiology, leveraging symptomatic search queries for the purpose of syndromic tracking of influenza-like illnesses (Ginsberg et al., 2009). The specific problems with GFT have been well described and discussed (Cook et al., 2011; Olson et al., 2013; Lazer et al., 2014), but the larger problem with GFT was the private ownership of the underlying data, which meant that the algorithm could not be reproduced and investigated independently. In other words, the public health community had no deep insight into the algorithm and the underlying data, was not able to directly contribute to its improvement, and had no say in the decision to shut down the system. It is understandable that no public health authority would be particularly keen on using such a black box system over which it has absolutely no control. However, the implementation of digital epidemiology into the daily workflow of public health authorities is perhaps the key goal of digital epidemiology in the future. Therefore, the field needs to focus on finding ways to make data openly accessible, at least to health authorities and researchers, and ideally to the community at large. This, however, is at odds with the current trend of major internet services substantially reducing the access to data. For example, Instagram, which is part of Facebook Inc. since 2012, has severely restricted access with an API update on June 1, 2016. How long Twitter data, a major digital epidemiology data source, will remain openly accessible is anyone’s guess, but is ultimately in the hands of the owners of Twitter Inc.

There are multiple responses to this trend, of which I will highlight two. The first response, ironically, is to do less digital epidemiology in the strict sense, namely to rely less on third-party data that was not generated for epidemiological purposes, and for the public health system to generate their own digital data streams that do not exclusively depend on corporate actors. Academic labs could play an important role in this development by creating the prototype systems, which, once their usefulness is established, could be supported, extended and maintained by public health authorities. Existing successful case studies of innovative approaches coming out of academic labs include HealthMap (Brownstein et al., 2008) and InfluenzaNet (Paolotti et al., 2014), and I expect to see many more in the near future. The second response is to build on the increasing legal reality that data generated by individuals, no matter through which corporate service, belongs to the individuals who generated it - or that, at least, the individual has a right to a copy of his or her data. For example, the EU General Data Protection Regulation, taking effect on May 25, 2018, states in Article 20 that a "data subject shall have the right to receive the personal data concerning him or her, which he or she has provided to a controller, in a structured, commonly used and machine-readable format and have the right to transmit those data to another controller without hindrance from the controller to which the personal data have been provided" http://eur-lex.europa.eu/legal-content/en/TXT/?uri=CELEX%3A32016R0679. It is conceivable that a representative fraction of the population could be convinced to share health-related data with public health authorities, either directly or through third parties such as health cooperatives.

Beyond the data-related trends, another major trend is concerned with the analytics of the data, rather than the data itself, and can be captured by the term “machine learning”. Machine learning, broadly defined as the ability for computers to learn patterns from data without being explicitly programmed, has seen enormous developments in the past decade, particularly also in the subfields of image recognition and natural language comprehension. The breakthroughs in these subfields were themselves mostly realized using a branch of machine learning called deep learning (LeCun et al., 2015), which builds on artificial neural networks that can be constructed in such a way as to learn rapidly from large amounts of data to correctly map an input (such as an image or a sentence) to an output (such as a diagnosis or a sentiment). Recent high-profile examples in the health domain include the demonstration that a deep learning algorithm trained on almost 130,000 clinical images of skin lesions performed on par with 21 board-certified dermatologists when performing skin cancer classification (Esteva et al., 2017). Such demonstrations now appear almost daily in the scientific literature, underlying the rapidly growing interest in this field, and artificial intelligence (AI) in general. The fact that these large neural networks are currently mostly black boxes whose inner workings are poorly understood is often leveled as a major criticism against them. However, this criticism may not be valid for very long, as substantial resources are currently invested towards a better understanding of how exactly they work. Overall, given these developments, I expect machine learning to be the major trend dominating digital epidemiology in the next decade.

Interestingly, the deep learning approach itself is currently highly dependent on the availability of very large datasets from which neural networks can learn. Thus, in order to reap the benefits from this technology, one needs access to large datasets. The need for large, open datasets for digital epidemiology will therefore only continue to grow. Due to the strong open source software movement particularly also from academia, most of the algorithms underlying deep learning are openly and freely available. The same is not generally true for data, and access to large, high quality datasets is thus becoming the limiting factor in the development of AI. In my view, it is therefore in the interest of the public to ensure that as much data as possible is openly accessible. This collective interest is clearly at odds with the understandable desire of most individuals to have as little personal data as possible publicly accessible in order to protect their privacy. There is no straightforward solution to the challenge of this “collective vs individual” conflict of interest. Data cooperatives with restricted access as described above may point the way towards a possible path forward. Another important development is homomorphic encryption (Gentry, 2009), a method by which computations can be carried out on encrypted data. This method currently still has relevant speed limits, but given the improvements in the past few years (Dowlin et al., 2016) these limits are likely to become irrelevant in the very near future, offering a potentially extremely interesting way to resolve the collective vs individual conflict of interest in open data.
